# CEUS-Based Radiomics Can Show Changes in Protein Levels in Liver Metastases After Incomplete Thermal Ablation

**DOI:** 10.3389/fonc.2021.694102

**Published:** 2021-08-26

**Authors:** Haiwei Bao, Ting Chen, Junyan Zhu, Haiyang Xie, Fen Chen

**Affiliations:** ^1^Department of Ultrasound, The First Affiliated Hospital, Zhejiang Chinese Medical University, Hangzhou, China; ^2^Key Laboratory of Combined Multi-organ Transplantation, The First Affiliated Hospital, Zhejiang University, Hangzhou, China

**Keywords:** CEUS (contrast-enhanced ultrasound), radiomics, machine learning, liver metastases, thermal ablation

## Abstract

**Objective:**

To investigate the ability of contrast-enhanced ultrasound (CEUS)-based radiomics combined with machine learning to detect early protein changes after incomplete thermal ablation.

**Methods:**

HCT-26 colorectal adenoma cells were engrafted into the livers of 80 mice, which were randomly divided into 4 groups for palliative laser ablation. Changes in heat shock protein (HSP) and apoptosis-related protein expression in the tumors were assessed. SCID mice subjected to CEUS and ultrasonography were divided into training (n=56) and test (n=24) datasets. Then, 102 features from seven feature groups were extracted. We use the least absolute shrinkage and selection operator (LASSO) feature selection method to fit the machine learning classifiers. The feature selection methods and four classifiers were combined to determine the best prediction model.

**Results:**

The areas under the receiver-operating characteristic curves (AUCs) of the classifiers in the test dataset ranged from 0.450 to 0.932 (median: 0.721). The best score was obtained from the model in which the omics data of CEUS was analyzed in the arterial phase by random forest (RF) classification.

**Conclusions:**

A machine learning model, in which radiomics characteristics are extracted by multimodal ultrasonography, can accurately, rapidly and noninvasively identify protein changes after ablation.

## Introduction

Whether liver metastases can be inactivated in patients with liver metastases from colorectal cancer (CRLM) is a key issue influencing the survival and long-term tumor-free survival of patients (1–3). Thermal ablation has been considered an effective method in the treatment of such patients, but sometimes the residual tumor cells cannot be completely inactivated by ablation (4, 5). Various imaging studies have a good judgment on the necrotic changes at the histological level after ablation and have formed certain clinical guidelines, but there are few reports on the changes at the molecular level, especially at the protein level. The molecular changes before and after ablation and the combination of molecular targeted therapy on this basis are the current research focus (6–8). At the same time, there is an urgent need for noninvasive imaging methods to reveal the molecular changes in the tumor before and after ablation (9).

The development of imaging has enabled the successful transformation of high-dimensional medical images into massive amounts of multilevel quantitative data (10, 11). In theory, imaging has the potential to reveal the tumor phenotype and molecular changes from the level of macroscopic characteristics of organ tissues to the level of local cell and molecular characteristics (12). The accurate, timely and sensitive display of tumor molecular characteristics by imaging is of great significance for treatment (13–16). CT, MRI and PET have been successfully explored in these fields (17–20). There are relatively few reports on multimodal ultrasound, including contrast-enhanced ultrasound (CEUS), and there is no standard for omics exploration methods (21–23). The main difficulty is the lack of universal ultrasound omics analysis methods, especially for CEUS data (24, 25).

Multimodal ultrasound in the imaging-based diagnosis of liver metastases has the advantages of being easy, repeatable, nonradioactive, and highly sensitive and is the main imaging means for guiding thermal ablation. In most interventional treatment centers in China, the rate at which ultrasound can detect liver metastases is an important factor for decision-making regarding ablation. We explored whether CEUS-based multimode ultrasound imaging can detect early molecular changes in incomplete tumor ablation in animal models.

## Materials and Methods

### Animal Model

**Figure 1:**
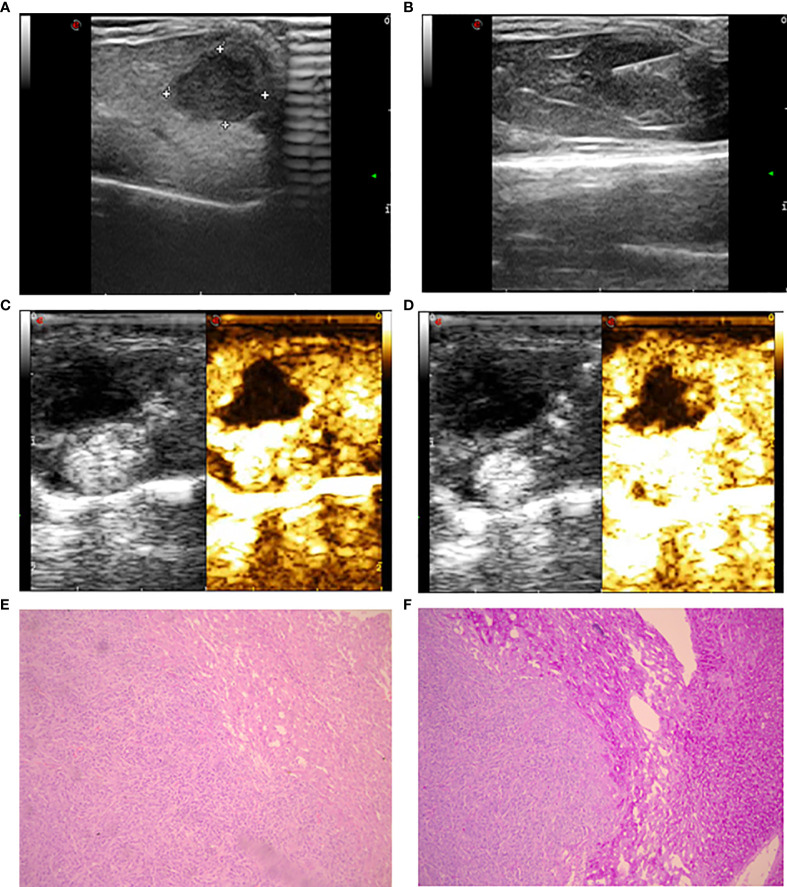
Ultrasonic image and histopathology of the implanted tumor. **(A)** Gray ultrasound showed hypoechoic metastases in the liver parenchyma with clear boundaries. **(B)** Ultrasound showed laser fiber into the center of the tumor. **(C)** The 5-second image of contrast-enhanced ultrasound in liver metastases. **(D)** The 25-second image of contrast-enhanced ultrasound in liver metastases. **(E)** The morphology of intestinal adenocarcinoma cells was uniform with large and deep stained nuclei (HE staining). **(F)** Compared to intratumoral tissue, there was increased staining in liver tissue with clear edges (periodic acid-Schiff staining).

All experimental procedures followed Zhejiang University Laboratory Animal Operating Regulations. (http://www.lac.zju.edu.cn/cms/12997). This research protocol was approved by the Research Ethics Committee of the First Affiliated Hospital of Zhejiang University. The severe combined immunodeficiency (SCID) mice were housed in a specific pathogen-free (SPF) animal room and were underwent a 12-h light/dark cycle to obtain free food and tap water. The temperature of the room is 20-25°C and the humidity is 50-60%. The 10mm long HCT-26 colorectal adenoma tumor tissue was cut into 2-mm3 pieces and implanted into the left lobe liver of SCID mice by surgical incision. According to the different ablation conditions, a total of 80 mice were divided into 4 groups with 20 mice in each group. The grouping was performed with a random number method. The first group is a blank control group, the second group is a sham puncture group, the third group and the fourth group are incomplete ablation groups. The incomplete ablation experiments were performed after 2 weeks when tumors had grown to an average diameter of 0.6-0.7 cm (**Figure 1A**). The SCID mice were killed 18 h after incomplete ablation, and the largest tumor diameter was 0.8-1.0 cm.

### Palliative Ablation Method

Intraperitoneal anesthesia was administered to the SCID mice with approximately 200 μl 4% chloral hydrate at a dose of 400 mg/kg mouse weight. We applied the conditions established in the previous study to construct an incomplete ablation model (26, 27). Mice were fixed on the operating table with local disinfection. An ultrasound-guided 21-G percutaneous transhepatic cholangiography (PTC) needle was used to puncture the tumor. After confirming the position of the needle by high-frequency ultrasound, the operator removed the inner core and inserted a laser fiber into the needle (Mylab™ Twice, Esaote SpA) (**Figure 1B**). After confirming that the fiber was positioned at 1/3 of the length of the tumor, the power was set at 1 W, and the foot switch was activated for continuous laser ablation; the treatment time was 0 seconds, 30 seconds and 60 seconds (total dose of 0 J, 30 J, 60 J). Local hemostasis was conducted by applying gentle pressure following treatment.

### Multimodal Ultrasound Examination and Image Evaluation

All ultrasound examinations were performed by two experienced radiologists who had more than 10 years of experience in ultrasound-guided interventional procedures. Conventional sonography was conducted with MyLab Twice (Esaote SpA, Genoa, Italy) with a linear array transducer with a frequency range of 18-22 MHz. Scans were performed under the abdominal preset, and the imaging focal zone was positioned posterior to the level of the lesion. For the target lesion, the largest section in terms of both longitudinal and transverse views was stored. Color Doppler of each lesion was performed by using the same transducer, and the picture was recorded.

The linear array transducer with a frequency range of 1.0 to 4.0 MHz is equipped with a contrast-specific contrast pulse sequencing imaging mode for CEUS inspection. Contrast-enhanced sonography was performed using SonoVue (Bracco SpA, Milan, Italy), a second-generation ultrasound contrast agent consisting of microbubbles of sulfur hexafluoride gas. A 0.1-mL bolus of SonoVue was hand injected through a 25-G intravenous catheter in the caudal vein. The target lesion in the largest plane was continuously observed and documented with a 60-second long clip (**Figures 1C, D**). All the above mentioned ultrasound examinations were completed on the experimental day 8 h after incomplete ablation or sham puncture.

CEUS features (including the enhancement level, enhancement homogeneity, enhancement boundary, and feeding artery) were evaluated and recorded. All digital cine clips of the study population were retrospectively reviewed by two investigators (C.F. and B.H.W.), each of whom has more than 10 years of experience in evaluating liver CEUS scans. They were asked to evaluate and record the imaging features of all the mice using a standardized approach. In cases of discordance, a consensus reading was performed, and the classification judgment was made.

### Sample Collection and Molecular Biological Examination

The SCID mice were euthanized *via* intraperitoneal injection of 2% sodium pentobarbital at a dose of 150 mg/kg body weight. Cervical dislocation was used to confirm the death of 80 mice 18 h post-operation. The 80 tumors were cut along their diameter, and one-quarter was fixed in 4% paraformaldehyde for 48 h at room temperature, whereas the remaining three-quarters of each tumor was kept in a liquid nitrogen jar. Histopathology was performed with HE and periodic acid-Schiff (PAS) staining (**Figures 1E, F**).

Terminal deoxynucleotidyl transferase dUTP nick end labeling (TUNEL) was used to determine the apoptosis of the tumor cells in the liver that underwent incomplete ablation. For each section, five or more high-magnification fields (200x) with at least 500 cells were selected to count the number of cells emitting green fluorescence and to subsequently calculate the apoptosis index (Ai). The equation was as follows: Ai = (number of positive cells/number of total cells) 100% ± SD.

Small tumor samples were lysed on ice using RIPA lysis buffer (P0013B, Beyotime, China) containing a protease inhibitor cocktail; cellular debris was pelleted by centrifugation at 12,000 rpm for 5 min at 4°C, and the supernatants were harvested. Protein concentrations were measured by a bicinchoninic acid (BCA) protein assay kit (P0010 Beyotime, China). The protein lysates (30 µg per lane) were separated *via* SDS-PAGE. Following separation on an 8%~15% acrylamide gel, the proteins were transferred onto PVDF membranes. The membrane containing the proteins was successively incubated with blocking buffer (overnight at 4°C), with a primary antibody (37°C for 1 h) and with a secondary antibody (37°C for 1 h). The following primary antibodies were used: anti-Bax (1:3000; ab32503), anti-caspase-3 (1:1000; ab184787), anti-Hsp70 (1:1000; Ab2787), anti-Hsp90 (1:1000; Ab13492), and anti-GAPDH (1:1000; ab181602), all from Abcam Biotechnology. HRP-conjugated goat anti-mouse IgG H&L (1:3000; SE131, Solarbio, USA) was used as the secondary antibody. Chemiluminescence detection was achieved by exposure to film in a darkroom. Following development and fixation with washing buffer at 20−25°C for 10 min (P0019, Beyotime Institute of Biotechnology), the film was visualized with an enhanced chemiluminescence system (ECL, Beyotime, China). The densities of the protein bands were determined using ImageJ software (v1.46; National Institutes of Health), normalized to actin expression and quantified using Microsoft Excel software (version 2016, Microsoft Corporation).

### Feature Extraction and Selection

**Figure 2:**
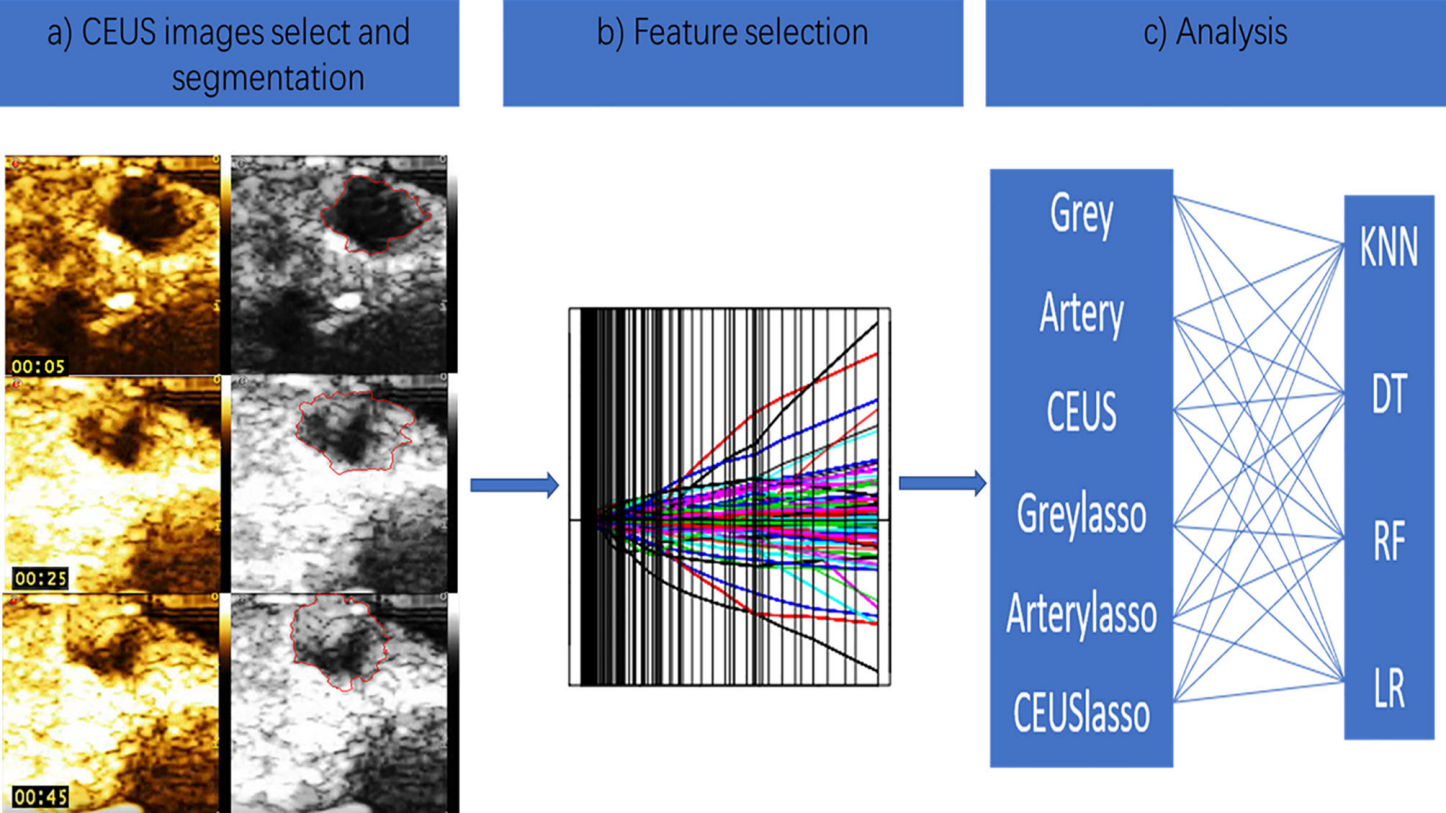
The CEUS-based radiomics analysis schematic. **(A)** The CEUS images of mouse was extracted at 5,25 and 45 seconds. **(B)** Lasso regression was used as feature selection method. **(C)** Four supervised machine learning algorithms were applied.

Dynamic CEUS was used to obtain a series of static images at a frequency of one per second. All stored images were transformed into an 8-bit bitmap. For each lesion, a region of interest (ROI) around the tumor border was delineated on the largest cross section with the ABsnake plugin using ImageJ software semiautomatically. Two doctors porformed three times combined with their own judgment. Then, a total of 102 features were extracted from the ROI using PyRadiomics (version 1.3.0; Computational Imaging and Bioinformatics Lab, Harvard Medical School). The CEUS images of each mouse were extracted at 5, 25, and 45 seconds (**Figure 2A**). To reduce the differences in semi-automatic manual segmentation between sonographers, we calculated the intraclass correlation coefficient (ICC) of each feature between two doctors. We extracted the following three types of data for machine learning analysis: the grayscale data, contrast-enhanced arterial phase data (15 seconds), and the whole course of CEUS data. The absolute values of the differences in the 5- and 25- second and the 25-second and 45-second CEUS radiomics data per mouse were added as the data representing the whole course of CEUS for subsequent analysis. To construct nonredundant and robust combined radiomic signatures, the least absolute shrinkage and selection operator (LASSO) regression method was used (**Figure 2B**). The complexity of the LASSO regression is controlled by a tuning parameter lambda (λ) with the rule that as the value of λ increases, the penalty for each variable coefficient also increases. Only nonzero coefficient variables were selected in this method.

### Machine Learning and Model Performance Evaluation

Data analysis and training of binary classifiers was performed using the Python programming language (Python Software Foundation, version 3.7.4, 2019, available at http://www.python.org), including the packages “numpy,” “pandas,” “sklearn,” and the other package and the Anaconda integrated development environment (version 1.9.12, Anaconda Inc., USA).

We applied 4 supervised machine learning algorithms; these classifiers were k nearest neighbors (KNN),decision tree (DT), random forest(RF) and logistic regression (LR) (**Figure 2C**).

There were six kinds of radiomics data as the inputs of each machine learning model(1. Grayscale ultrasound 2. Contrast-enhanced arterial phase data 3. The whole course of CEUS 4. Grayscale data with LASSO regression 5. Contrast-enhanced arterial phase data with LASSO regression 6. The whole course of CEUS with LASSO regression).These 4 classification methods combined with six kinds radiomics data to establish 24 (6× 4 = 24) models.

Each of the 24 models was trained and 10-fold cross validated in the training set with scikit-learn. The receiver operating characteristic (ROC) curve and area under the ROC curve (AUC) were employed to evaluate the predictive accuracy of the radiomics signatures developed. The model that had the highest AUC value in the test dataset was selected as the final model.

A two-sided p value of < 0.05 was used as the criterion to indicate a statistically significant difference. Statistical analysis was conducted with SPSS 22.0 for Windows (Chicago, IL).

**Figure 3:**
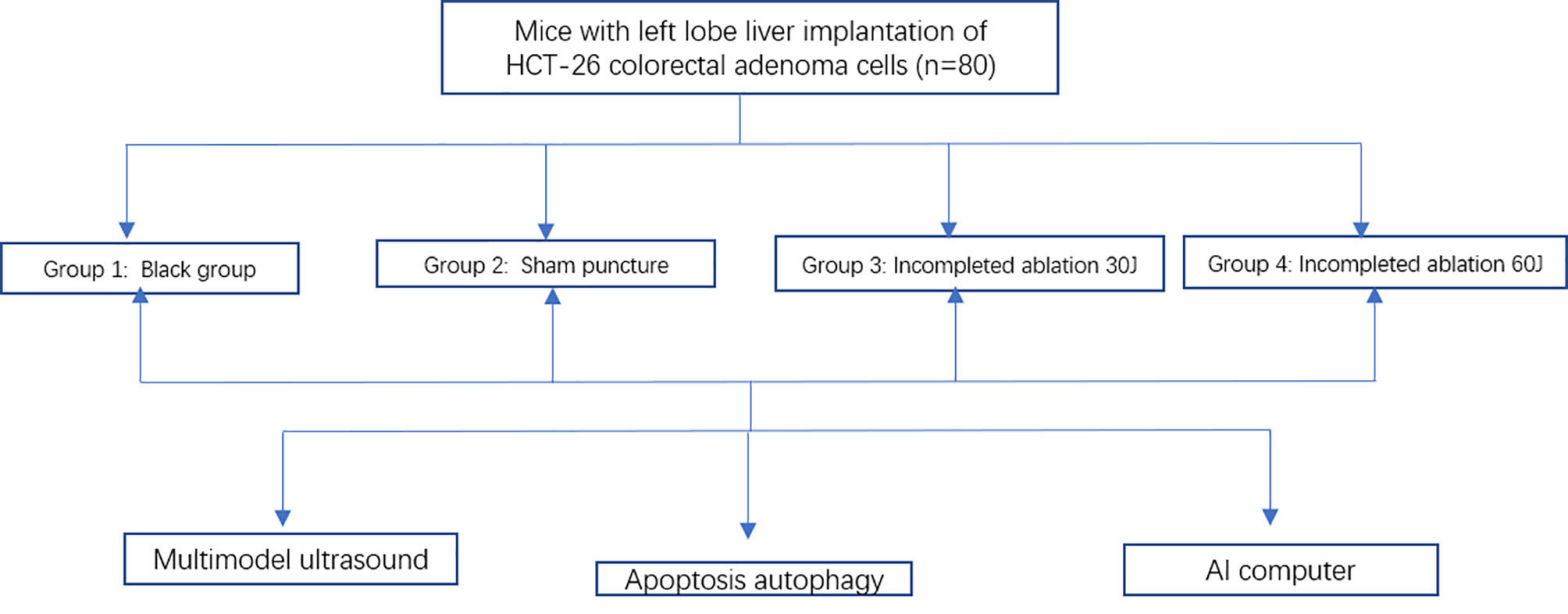
The flow chart shows the three main steps process in our study.

The above is the main process of this study (**Figure 3**).

## Results

### Molecular Changes After Tumor Ablation

#### The Expression of HSPs Is Increased After Ablation

**Figure 4:**
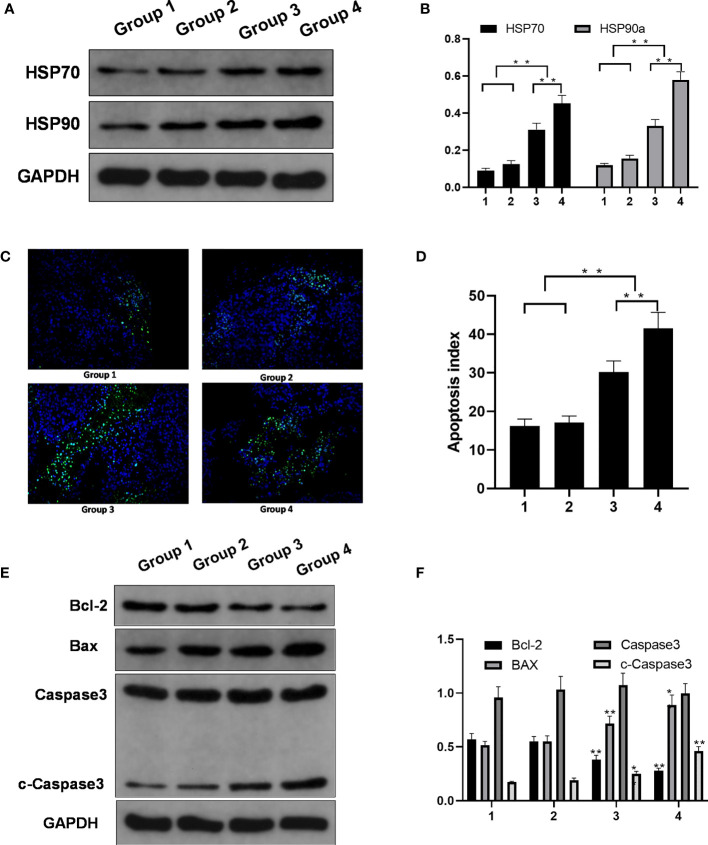
Molecular changes in the control groups and the experimental groups. **(A)** HSP70 and HSP90a expression. **(B)** HSP70 and HSP90a expression levels in four groups. Significant differences were calculated in groups 1 and 2 *vs*. groups 3 and 4, groups 3 *vs*. 4. **(C)** TUNEL expression (magnification). **(D)** Apoptosis index of the four groups. Significant differences were calculated in groups 1 and 2 *vs*. groups 3 and 4, groups 3 *vs*. 4. **(E)** Protein expression. **(F)** Bcl-2, BAX, and c-capase3 protein expression level in four groups. Significant differences were calculated between groups 1 and 2 *vs*. groups 3 and 4. *p < 0.05, **p < 0.01.

Compared with those in groups 1 and 2, the expression levels of two HSPs (HSP70 and HSP90a) were increased in groups 3 and 4. The western blotting results confirmed that the HSP70 and HSP90a expression levels were significantly higher in experimental groups 3 and 4 than in control groups 1 and 2; the relative HSP70 and HSP90a expression levels in group 4 were higher than those in group 3 (p < 0.01) (**Figures 4A, B**).

#### Apoptosis in the Incomplete Ablation Group and the Control Group

The apoptosis rate was higher in groups 3 and 4 than in groups 1 and 2. The TUNEL assay showed more green fluorescence accumulation in groups 3 and 4 than in groups 1 and 2. Ai was higher for the tumors in groups 3 and 4 than for the tumors in groups 1 and 2, and Ai was higher in group 4 than in group 3 (**Figures 4C, D**).

The expression of the apoptosis protein Bcl-2 was lower in groups 3 and 4 than in groups 1 and 2. The expression of the apoptosis protein BAX was higher in groups 3 and 4 than in groups 1 and 2. The relative expression levels of cleaved caspase-3 protein detected by western blot analysis showed were significantly different between groups 1 and 2 *vs*. groups 3 and 4 (**Figures 4E, F**).

### Interobserver Agreement

The interobserver reproducibility of feature extraction by the two ultrasonographers was good, with ICCs ranging from 0.789 to 0.932. Therefore, all the outcomes were based on the measurements made by the first sonographer.

### Feature Selection

The training dataset includes 102 radiomics features. The features were grouped into first-order statistics (18 features) and shape-based (9 features), gray level dependence matrix (GLDM, 14 features), gray level cooccurrence matrix (GLCM, 24 features), gray level run length matrix (GLRLM, 16 features), gray level size zone matrix (GLSZM, 16 features), and neighboring gray tone difference matrix (NGTDM, 5 features) features. A radiomics signature was further constructed based on the 102 features with respective nonzero coefficients selected through the LASSO regression method.

### Model Performance Evaluation

**Figure 5:**
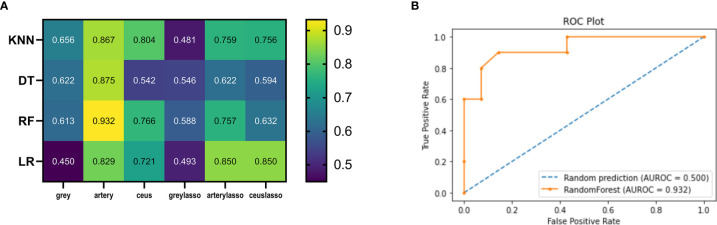
Heatmaps illustrating the predictive performance (AUC) of different combinations of feature selection methods (rows) and classification algorithms (columns). **(A)** Cross-validated AUC values of 24 models on the training and validation datasets. **(B)** ROC curve of RF_artery.

LR is one of the most commonly used generalized linear mixed models (GLMMs) for two classifications of data. The number of key features selected for building a radiomics signature was determined by the concordance index (C-index) value using 10-fold cross-validation. LR with L1 regularization had an AUC of 0.450-0.829 in the test dataset; LR with LASSO feature selection had an AUC of 0.493-0.850 in the test dataset (**Figure 5A**).

In the tune grid, we varied the max_depth between 1 and 10; min_impurity_decrease between 0-1; the best score was 0.792. The final model had a max_depth of 4 per decision tree. The final AUC of the DT classifier in the test dataset was 0.542-0.875. The final AUC of the DT classifier with LASSO feature selection in the test dataset was 0.546-0.622 (**Figure 5A**).

The selected RF classifiers consisted of 28 decision trees. In the tune grid, we varied the number of features randomly selected for each tree between 1 and 10. At a max-depth of 4, a plateau was observed at an AUC of 0.79, and the accuracy cannot be improved by increasing the tree depth. Consequently, the depth of each decision tree of the final model is 4 randomly selected predictors. The final AUC of the RF classifier was 0.613-0.932. The final AUC of the RF classifier with LASSO was 0.588-0.757 (**Figure 5A**).

We kept a KNN classifier as a reference because it is frequently applied, easy to implement, and robust, although its performance is worse than other classifiers. The number of neighbors used for classification varied from 1 to 11, and gradually increased by 1 during training. The best performance was achieved by the classifier using k = 1 neighbors. The final AUC of the KNN classifier was 0.656-0.867. The final AUC of the KNN classifier with LASSO was 0.481-0.759 (**Figure 5A**).

In **Figure 2A**, the mean AUCs of the 8 models are presented in the heatmap. AUCs ranged from 0.450 to 0.932; the median value was 0.721, and the best score was 0.932 (the RF model in the artery dataset; RF_artery) (**Figure 5B**). The AUCs of grayscale data combined with all the four classifiers ranged from 0.450-0.656 and the AUCs of graylasso data combined with all four classifiers ranged from 0.481-0.588. This shows that the classification value of grayscale ultrasonic image data is not high.

The AUC for the image identification ability of sonographers using conventional analytical methods is 0.705. The best machine learning score was significantly better than the ultrasonographer score (P<0.01).

## Discussion

In this preclinical radioproteomics study, we hypothesized that there was a causal relationship between image features and protein expression. To prove our hypothesis, we first employed a model of metastatic cancer implantation in SCID mice and performed incomplete ablation intervention. Our previous study showed that 30J and 60J laser ablation caused incomplete ablation, which induced increased apoptosis of the tumor tissue without causing significant necrosis (26, 27). Heat shock protein(HSP) family is sensitive to heat injury. Increased reactivity of HSP90 and HSP70 after incomplete thermal ablation has been reported in the literature (28, 29). The increase in HSP70 and HSP 90 indicates that the tumor tissue has suffered the corresponding thermal damage. Apoptosis related examinations including the increased expression of TUNEL, BAX protein, cleaved caspase-3 protein and the decrease of Bcl-2 protein, all proved the changes in apoptosis related proteins in tumor tissue (30).The related indicators—HSPs and apoptosis -related proteins—were assessed to confirm the ability of the model to detect protein level changes in a short time after incomplete ablation. The results show that the binary classification machine learning model trained by quantitative radiomics ultrasound data can distinguish changes in protein levels after incomplete ablation.

Radiomics has facilitated some meaningful advances in the field of liver tumor ablation (31). A total of 647 radiologic features of three-phase contrast-enhanced CT within 2 weeks before ablation were extracted from 184 HCC patients. LASSO Cox regression model was used to select valuable indexes. A recurrence prediction model was established based on clinicopathological factors and radiological features The results indicated that among the four radiomic models, the portal venous-phase model performed best in the validation subgroup (C-index = 0.736 (95% confidence interval [CI]: 0.726-0.856)). The predictive ability of the combined clinicopathological and radiological features model was significantly better than that of the simple clinical model (ANOVA, P < 0.05). 0001).

At present, there are several kinds of ultrasound contrast agents for monitoring microcirculation perfusion. Only SonoVue(Bracco Italy) has obtained the approval and license for abdominal imaging procedures in China. SonoVue is a stable sulfur hexafluoride microbubble surrounded by a phospholipid shell with a mean diameter of 2.5–6 μm (32). Guidelines and clinical practice recommendations for CEUS have been developed. CEUS is helpful to improve the detection of liver lesions. It has been suggested in planning and monitoring of liver metastatic tumor ablation.

CEUS with SonoVue is close to the gold standard of contrast-enhanced imaging in evaluating the short-term ablation rate (1-3 months) after ablation. It has the advantage of real-time and quick evaluation after ablation (immediate and 30 min), which can immediately guide re-ablation and improve the complete ablation rate of single ablation. In China and other countries, more than 50% of patients choose multimodal ultrasound guidance (33, 34). Early multimodality ultrasound has the advantage that incomplete ablation is detected sooner but brings about the challenge of interpreting dynamic processes such as transient edema. Therefore, it is valuable to explore the imaging omics evaluation of ablation based on ultrasound and CEUS immediately after surgery. Based on our limited knowledge, we have not yet seen a study assessing the protein level before and after tumor ablation by ultrasound imaging omics based on CEUS data. Only one study applied a radiomics technique with CEUS to assess tumor heterogeneity (35). In three tumor models of xenografted mice, the morphological and functional characteristics of tumor vessels were extracted by CEUS, and the tumor phenotype was classified by a trained linear support vector machine (SVM) incorporating features including the image intensity median, gray level cooccurrence matrix energy, vascular network length, and run length nonuniformity of the gray run matrix. The fourfold cross validation scheme was used to train the model, and a correct classification rate of 82.1% (95% CI: 0.640-0.92) was obtained. However, the author used an uncommon and hard-to-obtain vessel segmentation algorithm to extract CEUS radiomics data.

In this study, we successfully extracted the static radiomics data of grayscale ultrasound and CEUS at a certain time point by using the general open-source radiomics software PyRadiomics. The numerical differences between three static time CEUS scans were calculated to represent the whole process of CEUS. The data in this study are two-category small sample data, which are not suitable for deep learning methods that require large amounts of data, such as neural networks. We have selected the following four as exploration methods in machine learning classification algorithms. First, LR is one of the most commonly used methods for the two types of data classification, and traditional statistical methods can also be implemented. The KNN classifier is selected as a reference because it is a frequently used and easy to implement. The DT classifier and RF classifier are the most effective classification methods in machine learning. The results also prove that in this study, the effect of RF classification is the best, which has exceeded that of experienced doctors. The four most commonly used machine learning methods were applied to analyze six kinds of radiomics data. The RF_artery model proved to have the highest AUC value of 0.932. The value of CEUS in the arterial phase at 5 seconds was the highest, which is consistent with our clinical experience. The arterial phase of CEUS is often the most representative feature of microcirculation perfusion in tumors. In the LI-RADS classification standard of the liver contrast-enhanced ultrasound guide, the performance of the arterial phase of contrast-enhanced ultrasound occupies a high weight (36). The combination of anatomical and functional information might enable the development of better models for radiomics analysis.

In this study, relatively few animals were assessed, and a standardized acquisition protocol was used to image all mice. Thus, there is a risk that the data were overfitted and that the best image for analysis was not randomly selected for the test dataset. In addition, the small sample size makes the results more susceptible to data variability. Radiomics analysis can be influenced and challenged by the different types of scanners from different manufacturers in the clinical setting. This study provides good confidence that CEUS data can be used to perform radiomic analysis. Future studies should address the above mentioned issues.

## Conclusions

We have shown in *in vivo* preclinical models that radiomics is able to quantify early protein changes in tumors after incomplete ablation and identify differences that are not visible to the human eye. After incomplete ablation, the radiomics profile of CRLM appears to be different from that before surgery. These differences may be identified by binary classification algorithms, especially by RF trained with radiomics features extracted from the arterial-phase CEUS immediately after ablation, which showed the best AUC of 0.932, allowing for an early assessment of protein level changes after ablation. Further studies are needed to further validate its classification ability.

## Data Availability Statement

The original contributions presented in the study are included in the article. Further inquiries can be directed to the corresponding author.

## Ethics Statement

The animal study was reviewed and approved by Research Ethics Committee of the First Affiliated Hospital of Zhejiang University.

## Author Contributions

Conception and design: FC. Data acquisition: HWB, TC, JYZ, and FC. Data analysis and interpretation: FC and HWB. Drafting the article: FC and HWB. Revising the article: FC, HWB, TC, JYZ, and HYX. All authors contributed to the article and approved the submitted version.

## Funding

This work was funded by Zhejiang Province Basic Public Welfare Research Program (grant number LGD19C04007).

## Conflict of Interest

The authors declare that the research was conducted in the absence of any commercial or financial relationships that could be construed as a potential conflict of interest.

## Publisher’s Note

All claims expressed in this article are solely those of the authors and do not necessarily represent those of their affiliated organizations, or those of the publisher, the editors and the reviewers. Any product that may be evaluated in this article, or claim that may be made by its manufacturer, is not guaranteed or endorsed by the publisher.
